# Hypophosphatasia and the risk of atypical femur fractures: a case–control study

**DOI:** 10.1186/s12891-016-1191-8

**Published:** 2016-08-09

**Authors:** Timothy Bhattacharyya, Smita Jha, Hongying Wang, Daniel L. Kastner, Elaine F. Remmers

**Affiliations:** 1 Investigation performed at the National Institute of Arthritis and Musculoskeletal and Skin Diseases, National Institutes of Health, 10 Center Dr., Mail Code 1468, Bethesda, MD 20892-1150 USA; 2 National Institute of Child Health and Human Development (NICHD), National Institutes of Health (NIH), Bethesda, MD USA; 3 Inflammatory Disease Section, Medical Genetics Branch, National Human Genome Research Institute, National Institutes of Health, Bethesda, MD 20892-1849 USA

## Abstract

**Background:**

Case reports have linked adult hypophosphatasia as a possible cause of atypical femur fractures (AFF) associated with bisphosphonate use. Adult hypophosphatasia is an asymptomatic genetic condition which results in low alkaline phosphatase and elevated pyridoxal phosphate. We conducted a case–control study to assess the role of hypophosphatasia and atypical femur fracture.

**Methods:**

We recruited 13 control patients who took long term bisphosphonates without complication and 10 patients who sustained atypical femur fractures (mean bisphosphonate use, 9 years both cohorts). Patients underwent clinical exam and measurement of alkaline phosphatase and pyridoxal phosphate (PLP) levels. In addition, DNA was extracted and the *ALPL* gene was sequenced in both cohorts.

**Results:**

Low alkaline phosphatase levels (<55 U/L) were seen in 5/10 AFF patients and 5/13 control patients. Two control patients demonstrated low alkaline phosphatase levels and elevated PLP. The alkaline phosphatase (*ALPL)* gene exons and intron splice sites were sequenced in the atypical femur fracture and control cohorts and no coding mutations were identified in any subjects. Atypical femur fracture patients demonstrated more varus hip alignment (*p* < 0.048) with no significant difference in mechanical axis.

**Conclusions:**

We found no evidence of hypophosphatasia as a risk factor for atypical femur fractures. Laboratory findings of mildly low alkaline phosphatase activity were equally common in atypical and control cohorts and may be due to long term bisphosphonate use.

**Trial registration:**

Clinicaltrials.gov number NCT01360099. Prospectively registered May 20, 2011. First patient enrolled June 14, 2011.

## Background

Patients who take bisphosphonate medications long-term are at increased risk for atypical femur fractures [1]. The fractures are characterized by low energy injuries, prodromal pain, frequent bilaterally, and radiographic hallmarks of transverse orientation, minimal comminution, and lateral beaking [2]. There is a growing consensus that atypical femur fractures are due to long-term bisphosphonate medications causing suppressed bone turnover which results in inability of bone to repair microfractures [3] Varus hip geometry may increase the strain seen on the lateral cortex and increase the risk [4, 5]. However the vast majority of patients who take bisphosphonates experience a reduction in hip fracture risk and never experience an atypical femur fracture [6, 7] or show radiographic signs of an impending fracture even with 10 or more years of use [8]. The rarity of atypical femur fractures among bisphosphonate users suggests that certain people have an undiagnosed risk factor that predisposes them to atypical femur fracture when they are exposed to bisphosphonates.

Whyte [9] and Lane [10] have proposed an intriguing hypothesis that adult hypophosphatasia may be a risk factor for AFF. Hypophosphatasia is a generally asymptomatic genetic condition with low alkaline phosphatase activity seen in up to 5 % of adults. Clinical features include propensity to fracture, early loss of deciduous teeth, and pain. The disease is caused by mutations in the tissue non-specific alkaline phosphatase gene (TNSALP, current gene symbol, *ALPL*). The low alkaline phosphatase activity results in a buildup of pyridoxal 5′ phosphate (PLP) and inorganic pyrophosphate. Inorganic pyrophosphate is chemically similar to bisphosphonates and also inhibits bone mineralization. Patients with severe hypophosphatasia are known to sustain subtrochanteric fractures of the femur, and there has been a case report of atypical femur fracture in a patient with hypophosphatasia taking bisphosphonates [9].

We conducted a retrospective case–control study to test whether hypophosphatasia is a risk factor for atypical femur fracture.

## Methods

After IRB approval, we recruited from the community a cohort of patients who had sustained atypical femur fractures while taking bisphosphonates, and a control cohort of patients who had taken bisphosphonates for at least 5 years and not sustained a femur fracture. For the fracture cohort, we worked from a list of patients who had been referred to us after atypical femur fracture. Fourteen fracture patients were telephoned to participate in the study, and 4 declined. For the control cohort, we recruited patients who had previously enrolled in a longitudinal study of bisphosphonate users [8]. Thirteen patients were telephoned and 13 agreed.

Patients underwent a standardized history (including fracture history and dental history), a physical exam and standing long leg radiographs. Fasting blood was drawn and tested for alkaline phosphatase and PLP levels. The blood tests were performed at Mayo Medical Laboratories. Patients withheld vitamin supplementation for at least 1 week prior to testing because vitamin B ingestion can alter testing for PLP. In hypophosphatasia, alkaline phosphatase is low or low normal and PLP is elevated. Blood was drawn and DNA extracted for focused sequencing of the *ALPL* gene. Sequencing included the exons, part of the promoter, and the splice sites. We did not sequence the introns. Due to the cost of sequencing, we tested all of the atypical femur fracture patients, but only sequenced in the nine controls who manifested laboratory evidence of low or low normal alkaline phosphatase (below 60 ng/dl).

Statistical testing was done using Microsoft Excel and SPSS 10.0. Continuous variables were compared using the student’s *t*-test while chi-squared testing was performed for categorical variables. A post hoc power analysis was performed to assess the risk of type II error.

## Results

The cohort included ten atypical femur fracture patients and 13 asymptomatic controls. Age and gender distribution were similar in each cohort (Table 1). No patients described dental abnormalities such as early loss of deciduous teeth. All atypical femur fracture patients had sustained complete fractures and underwent intramedullary nailing. Atypical femur fracture patients had stopped taking bisphosphonates an average of 3.6 years. One patient in the fracture group and two in the control group were taking diuretics. The atypical group met at least four of the major diagnostic criteria (as required by ASBMR to meet the case definition) and zero to three of the minor diagnostic criteria [2].

**Table 1:** Demographic and Clincial Data

	Atypical femur fracture	Control	*P* value
Number of patients	10	13	
Mean age in years (range)	71 (68–79)	68 (62–84)	0.27
Female (%)	9/10 (90 %)	10/13 (77 %)	0.45
Bisphosphonate used			
Alendronate	7 (70 %)	10 (77 %)	0.68
Ibandronate	2 (20 %)	3 (23 %)	
Risendronate	1 (10 %)	0	
Proportion of patients with Vitamin D supplementation (%)	6/10 (60 %)	6/13 (46 %)	0.68
Mean years of bisphosphonate use (range)	9.2 (5–13)	8.8 (5–20)	0.79
Mean years since bisphosphonates stopped	3.6 (1–5)	2.0 (1–4)^a^	0.65
Mean alkaline phosphatase in U/L (range)^c^	58 (37–73)	56 (38–74)	0.81
Proportion of patients with abnormal alkaline phosphatase (below 50 U/L)	5/10 (40 %)	5/13 (38 %)	
Mean pyridoxal 5’ phosphate (PLP) in mcg/L^d^	29.8 (5–44)	20.6 (5–118)	0.37
Mean neck-shaft angle degrees (S.D.)	132 (3.1)	136 (5.2)	0.048
Mechanical axis angle degrees^b^ (S.D.)	−0.71 (5.0)	−0.15 (2.7)	0.76
Lateral distal femoral angle degrees (S.D.)	90.7 (5.0)	90.2 (2.7)	0.76

^a^10 of the thirteen patients had discontinued bisphosphonates. Three were continuing

^b^A positive number represents valgus alignment

^c^Normal range 55 to 105 U/L

^d^Normal range 5 to 50 mcg/L

Alkaline phosphatase levels were low in five of ten atypical femur fracture patients and were low in a five of 13 controls (*p = 0.58*). In the patients with low alkaline phosphatase levels, the confirmatory PLP test was elevated in only two control patients; both patients had a history of taking multivitamins regularly.

Genetic sequencing of the *ALPL* gene in patients with AFF and controls with low alkaline phosphatase showed no abnormalities which would have resulted in hypophosphatasia. We found only single nucleotide polymorphisms (SNPs) which are common variants in the population, and would not result in deleterious missense or nonsense mutation (Table 2). Polymorphisms were found equally in controls and atypical femur patients.

**Table 2:** Coding variants of the ALPL gene found in atypical fracture (AFF) patients and controls

Variant ID	cDNA variant	Protein variant	Variant allele frequency in AFF patients (*n* = 10)	Variant allele frequency in controls (*n* = 9)	Variant allele frequency in ESP-EA^a^
rs1780316	c.330 T > C	p.S110S	1.00	0.94	0.95
rs3200254	c.787 T > C	p.Y263H	0.20	0.17	0.11
rs3200255	c.876A > G	p.P292P	0.20	0.17	0.11
rs34605986	c.1565 T > C	p.V522A	0.10	0.00	0.10

^a^ *ESP-EA* NHLBI Exome-Sequencing Project-European American samples, *n* = 4300 samples

Review of standing long leg radiographs showed that atypical femur fracture patients had more varus hip geometry with a higher mean neck shaft angle than controls (136° versus 132°, *p* < 0.05). However this did not result in a difference in overall mechanical axis. The lateral distal femoral angle (normally 87°) was a mean of 90.7° in the atypical femur fracture cohort and 90.2° in the control cohorts (*p* = 0.70).

## Discussion

We found that patients who sustained an atypical femur fracture did not have laboratory findings or genetic mutations consistent with hypophosphatasia.

The initial case reports of atypical femur fractures resulted in numerous investigations. Several epidemiologic studies have documented the clear association between bisphosphonate use and atypical femur fracture [11, 12]. When the drug is withdrawn, the risk of fracture drops rapidly [13]. There is evidence of a dose response relationship [14]. Finally there is evidence that other bone anti-resorptive medications can produce atypical femur fractures [15].

However, the evidence indicates that bisphosphonate medications are the best first line treatment for osteoporosis, and are safe. Atypical femur fractures are rare, affecting one in 50,000 bisphosphonate users, and the fracture is readily treatable [9]. Furthermore, now that symptoms of prodromal thigh pain are recognized and the duration of treatment is being re-evaluated, the incidence of atypical femur fracture may be decreasing [16]. If the risk factors for atypical femur fracture could be elucidated, better targeting may eliminate atypical femur fractures entirely.

We investigated one proposed genetic risk factor for atypical femur fracture and found no mutations in the alkaline phosphatase gene. The hypophosphatasia hypothesis is intellectually attractive in that patients with severe hypophosphatasia are known to experience subtrochanteric femur fractures which are radiographically similar to atypical femur fracture, one case report suggests the possibility, and there is biological plausibility. However our data clearly demonstrate an absence of disease causing mutations in the *ALPL* gene. The low alkaline phosphatase levels we observed in nearly 50 % of AFF cases and non-AFF controls may simply be due to bisphosphonate treatment [17]. While many patients had discontinued medications, it well known that the half-life of bisphosphonates is almost 10 years, so prolonged effects are not unexpected [18].

It is possible that other genetic abnormalities predispose patients to atypical femur fracture. For example, a mutation in the genes for type I collagen may play a role because the fractures are on the tensile side of the femur. A logical approach to evaluate the genetic hypothesis would be to perform a genome-wide association study. However, in the absence of one or more variants with large effect size, a typical genome-wide association study requires about 1000 patients and a similar number of controls.

The main limitation of this study is the risk of Type II error. With a small sample size, there remains the possibility that an association between ALPL mutations exists but we did not detect it due to chance. A post hoc power analysis reveals that our study has 80 % power to detect a 40 % prevalence of ALPL mutations in atypical fracture patients. In other words, if the ALPL mutations were ten times more common (or higher) in atypical femur fracture patients, 80 % of studies of this size would detect a difference.

## Conclusions

We did not find laboratory or genetic evidence for hypophosphatasia in patients with atypical femur fracture. Further studies to define the biological risk factors for atypical femur fracture are warranted.

## Abbreviations

AFF, atypical femur fracture; ALPL, alkaline phoshosphatase gene locus; PLP, pyridoxal phosphate; SNPs, single nucleotide polymorphisms; TNSALP, tissue non-specific alkaline phosphatase gene
